# Supplementary material for: ERpred: a web server for the prediction of subtype-specific estrogen receptor antagonists
Source: PeerJ. 2021 Jul 9;9:e11716. doi: 10.7717/peerj.11716 (PMC8274494; doi:10.7717/peerj.11716)
Supplement: Supplemental Information 1 [file peerj-09-11716-s001.pdf]

Supplementary Information

ERpred: A web server for the prediction of subtype-specific estrogen receptor antagonists

Nalini Schaduangrat, Aijaz Ahmad Malik, Chanin Nantasenamat*

Center of Data Mining and Biomedical Informatics, Faculty of Medical Technology, Mahidol University, Bangkok 10700, Thailand

1. Top 10 active and inactive compounds of ERα

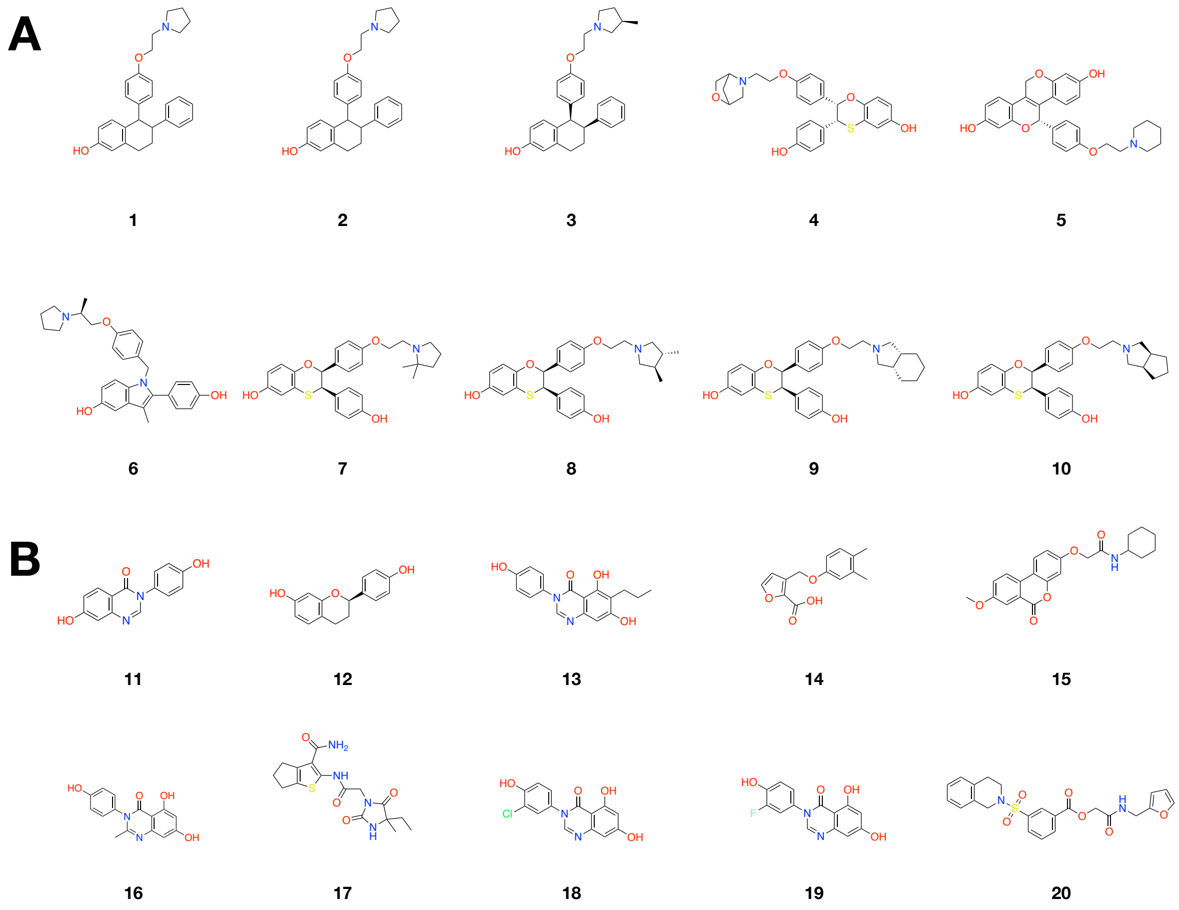

Supplementary Figure S1. Top 10 active and inactive compounds of ERα. Chemical compounds of the top 10 active (A) and inactive (B) compounds for ERα are shown. They are represented with numbers in parentheses while their corresponding IUPAC names are in text.

The set of ERα actives includes (4-(4-(4-chlorophenyl)piperazin-1-yl)phenyl)(6-hydroxy-2-(4 hydroxyphenyl)benzo[b]thiophen-3-yl)methanone (**1**), 6-phenyl-5-(4-(2-(pyrrolidin-1-yl)ethoxy)phenyl)-5,6,7,8-tetrahydronaphthalen-2-ol (**2**), (5R,6S)-5-(4-(2-((R)-3-methylpyrrolidin-1-yl)ethoxy)phenyl)-6-phenyl-5,6,7,8 tetrahydronaphthalen-2-ol (**3**), (2S,3R)-2-(4-(2-(2-oxa-5-azabicyclo[2.2.1]heptan-5-yl)ethoxy)phenyl)-3-(4-hydroxyphenyl)-2,3-dihydrobenzo[b][1,4]oxathiin-6-ol (**4**),
(S)-5-(4-(2-(piperidin-1-yl)ethoxy)phenyl)-5,11-dihydrochromeno[4,3-c]chromene-2,8-diol (**5**),
(S)-2-(4-hydroxyphenyl)-3-methyl-1-(4-(2-(pyrrolidin-1-yl)propoxy)benzyl)-1H-indol-5-ol (**6**),
(2S,3R)-2-(4-(2-(2,2-dimethylpyrrolidin-1-yl)ethoxy)phenyl)-3-(4-hydroxyphenyl)-2,3-dihydrobenzo[b][1,4]oxathiin-6-ol (**7**), (2S,3R)-2-(4-(2-((3R,4R)-3,4-dimethylpyrrolidin-1-yl)ethoxy)phenyl)-3-(4-hydroxyphenyl)-2,3-dihydrobenzo[b][1,4]oxathiin-6-ol (**8**),
(2S,3R)-3-(4-hydroxyphenyl)-2-(4-(2-((3aR,7aS)-octahydro-2H-isoindol-2-yl)ethoxy)phenyl)-2,3-dihydrobenzo[b][1,4]oxathiin-6-ol (**9**) and
(2S,3R)-2-(4-(2-((3aR,6aS)-hexahydrocyclopenta[c]pyrrol-2(1H)-yl)ethoxy)phenyl)-3-(4-hydroxyphenyl)-2,3-dihydrobenzo[b][1,4]oxathiin-6-ol (**10**).

The set of ERα inactive includes 7-hydroxy-3-(4-hydroxyphenyl)quinazolin-4(3H)-one (**11**), (R)-2-(4-hydroxyphenyl)chroman-7-ol (**12**), 5,7-dihydroxy-3-(4-hydroxyphenyl)-6-propylquinazolin-4(3H)-one (**13**), 3-((3,4-dimethylphenoxy)methyl)furan-2-carboxylic acid (**14**),
N-cyclohexyl-2-((8-methoxy-6-oxo-6H-benzo[c]chromen-3-yl)oxy)acetamide (**15**),
5,7-dihydroxy-3-(4-hydroxyphenyl)-2-methylquinazolin-4(3H)-one (**16**),
2-(2-(4-ethyl-4-methyl-2,5-dioxoimidazolidin-1-yl)acetamido)-5,6-dihydro-4H-cyclopenta[b]thiophene-3-carboxamide (**17**),
3-(3-chloro-4-hydroxyphenyl)-5,7-dihydroxyquinazolin-4(3H)-one (**18**),
3-(3-fluoro-4-hydroxyphenyl)-5,7-dihydroxyquinazolin-4(3H)-one (**19**) and
2-((furan-2-ylmethyl)amino)-2-oxoethyl 3-((3,4-dihydroisoquinolin-2(1H)-yl)sulfonyl)benzoate (**20**).

2. Top 10 active and inactive compounds of Erβ

A

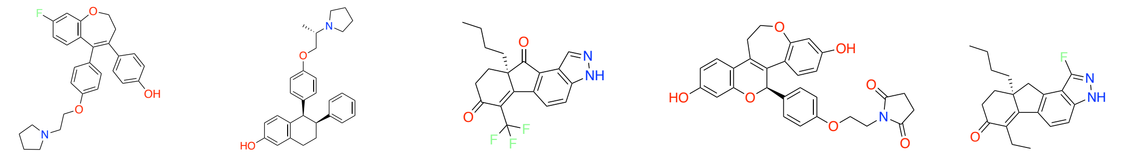

21 22 23 24 25

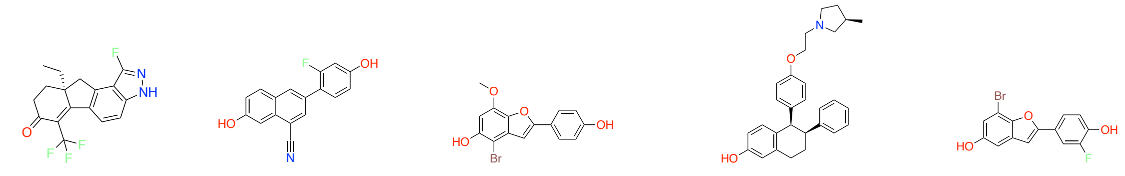

26 27 28 29 30

B

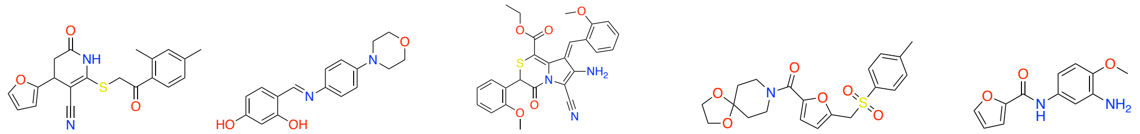

31 32 33 34 35

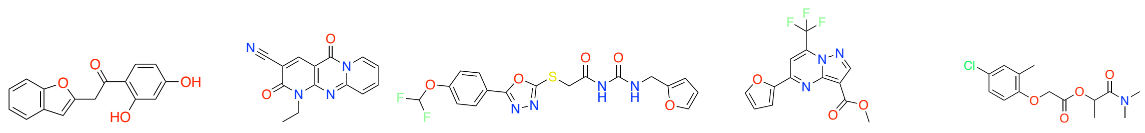

36 37 38 39 40

Supplementary Figure S2. Top 10 active and inactive compounds of ERβ. Chemical compounds of the top 10 active (A) and inactive (B) compounds for ERβ are shown. They are represented with numbers in parentheses while their corresponding IUPAC names are in text.

Chemical compounds of the top 10 active (A) and inactive (B) compounds for ERβ are shown in Supplementary Figure S2. They are represented with numbers in parentheses while their corresponding IUPAC names are as follows. The set of ERβ active includes 4-(8-fluoro-5-(4-(2-(pyrrolidin-1-yl)ethoxy)phenyl)-2,3-dihydrobenzo[b]oxepin-4-yl)phenol (**21**), (5R,6S)-6-phenyl-5-(4-((S)-2-(pyrrolidin-1-yl)propoxy)phenyl)-5,6,7,8-tetrahydronaphthalen-2-ol (**22**), (R)-9a-butyl-6-(trifluoromethyl)-9,9a-dihydroindeno[2,1-e]indazole-7,10(3H,8H)-dione (**23**), (R)-1-(2-(4-(5,11-dihydroxy-1,8-dihydro-2H-

benzo[2,3]oxepino[4,5-c]chromen-8-yl)phenoxy)ethyl)pyrrolidine-2,5-dione (**24**), (S)-9a-butyl-6-ethyl-1-fluoro-8,9,9a,10-tetrahydroindeno[2,1-e]indazol-7(3H)-one (**25**), (S)-9a-ethyl-1-fluoro-6-(trifluoromethyl)-8,9,9a,10-tetrahydroindeno[2,1-e]indazol-7(3H)-one (**26**), 3-(2-fluoro-4-hydroxyphenyl)-7-hydroxy-1-naphthonitrile (**27**), 4-bromo-2-(4-hydroxyphenyl)-7-methoxybenzofuran-5-ol (**28**), (5R,6S)-5-(4-(2-((R)-3-methylpyrrolidin-1-yl)ethoxy)phenyl)-6-phenyl-5,6,7,8-tetrahydronaphthalen-2-ol (**29**) and 7-bromo-2-(3-fluoro-4-hydroxyphenyl)benzofuran-5-ol (**30**).

The set of ERβ inactive includes 2-((2-(2,4-dimethylphenyl)-2-oxoethyl)thio)-4-(furan-2-yl)-6-oxo-1,4,5,6-tetrahydropyridine-3-carbonitrile (**31**), (E)-4-(((4-morpholinophenyl)imino)methyl)benzene-1,3-diol (**32**), ethyl(E)-7-amino-6-cyano-8-(2-methoxybenzylidene)-3-(2-methoxyphenyl)-4-oxo-3,4-dihydro-8H-pyrrolo[2,1-c][1,4]thiazine-1-carboxylate (**33**), (1,4-dioxa-8-azaspiro[4.5]decan-8-yl)(5-(tosylmethyl)furan-2-yl)methanone (**34**), N-(3-amino-4-methoxyphenyl)furan-2-carboxamide (**35**), 2-(benzofuran-2-yl)-1-(2,4-dihydroxyphenyl)ethan-1-one (**36**), 1-allyl-2,5-dioxo-1,5-dihydro-2H-dipyrido[1,2-a:2',3'-d]pyrimidine-3-carbonitrile (**37**), 2-((5-(4-(difluoromethoxy)phenyl)-1,3,4-oxadiazol-2-yl)thio)-N-((furan-2-ylmethyl)carbamoyl)acetamide (**38**), methyl 5-(furan-2-yl)-7-(trifluoromethyl)pyrazolo[1,5-a]pyrimidine-3-carboxylate (**39**) and 1-(dimethylamino)-1-oxopropan-2-yl 2-(4-chloro-2-methylphenoxy)acetate (**40**).
